# Supplementary material for: Regional genetic correlations highlight relationships between neurodegenerative disease loci and the immune system
Source: Commun Biol. 2023 Jul 15;6:729. doi: 10.1038/s42003-023-05113-5 (PMC10349864; doi:10.1038/s42003-023-05113-5)
Supplement: Supplementary file 2 — Supplementary Information [file 42003_2023_5113_MOESM2_ESM.pdf]

Supplementary Tables, Figures and Note 1

Supplementary Tables

Supplementary Table 1. Genome-wide genetic correlations between pairs of diseases evaluated in the study estimated with LD Score Regression. r_g = genetic correlation; SE = standard error. ns = non-significant correlations; • = nominal correlations (p-value < 0.05); ** = Bonferroni-corrected significant correlations (p-value < 0.0014).

GWAS trait 1	GWAS trait 2	r_g	SE	p-value	Significance
AD	ALS	0.19	0.089	0.0359	•
AD	CD	-0.13	0.053	0.0125	•
AD	FTD	0.29	0.291	0.3126	ns
AD	LBD	0.33	0.221	0.1324	ns
AD	MS	-0.06	0.059	0.2899	ns
AD	PD	0.23	0.089	0.0096	•
AD	SCZ	0.03	0.038	0.4934	ns
AD	UC	-0.08	0.066	0.2038	ns
ALS	CD	0.1	0.051	0.0618	ns
ALS	FTD	0.6	0.430	0.164	ns
ALS	LBD	0.5	0.208	0.0168	•
ALS	MS	0.03	0.060	0.6411	ns
ALS	PD	0.14	0.065	0.0334	•
ALS	SCZ	0.06	0.036	0.0722	ns
ALS	UC	0.1	0.055	0.0634	ns
CD	FTD	0.13	0.144	0.3724	ns
CD	LBD	0.1	0.109	0.3797	ns
CD	MS	0.15	0.047	0.001	**
CD	PD	0	0.040	0.998	ns
CD	SCZ	0.13	0.024	1.68E-07	**
CD	UC	0.62	0.033	2.43E-80	**
FTD	LBD	0.32	0.537	0.5546	ns
FTD	MS	-0.21	0.193	0.2745	ns

FTD	PD	0.48	0.285	0.0926	ns
FTD	SCZ	0.19	0.153	0.2196	ns
FTD	UC	0.17	0.184	0.3589	ns
LBD	AD	0.33	0.221	0.1324	ns
LBD	MS	-0.02	0.113	0.8685	ns
LBD	PD	0.65	0.196	0.001	**
LBD	SCZ	-0.03	0.075	0.6509	ns
LBD	UC	-0.01	0.130	0.9129	ns
MS	PD	0.03	0.045	0.472	ns
MS	SCZ	0.05	0.029	0.1197	ns
MS	UC	0.3	0.045	1.45E-11	**
PD	SCZ	0.02	0.030	0.5272	ns
PD	UC	0.05	0.049	0.2795	ns
SCZ	UC	0.16	0.028	1.90E-08	**

Supplementary Table 2. Number of genome-wide significantly expressed genes per cell type from the OneK1K single-cell eQTL data.

Cell Type	N significantly expressed genes
Classical monocytes	196
CD4+ naïve T cells	2,089
CD4+ effector memory T cells	543
CD8+ naïve T cells	930
CD8+ effector memory T cells	1,018
Naïve B cells	502
Memory B cells	383

Supplementary Figures

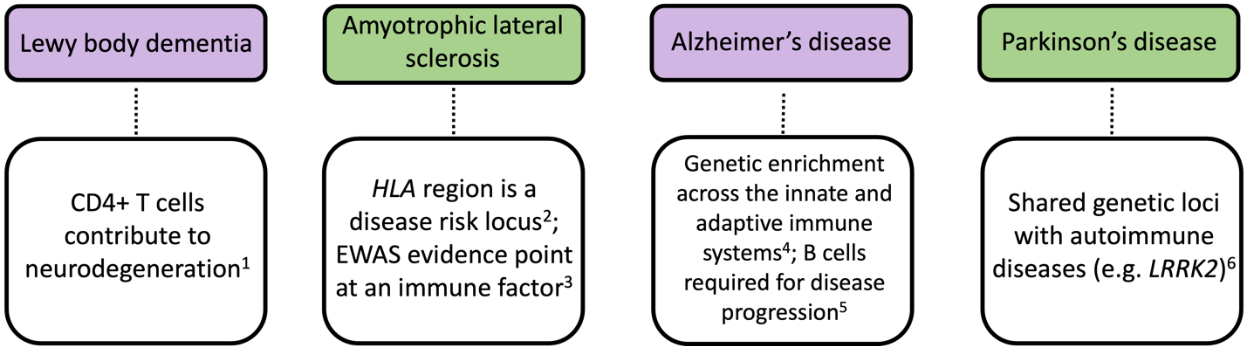

Supplementary Figure 1. Summary of computational and experimental evidence that have highlighted the involvement of the immune system in complex neurodegenerative diseases. [1] Gate et al. 2021 *Science ;* [2] van Rheenen et al. 2021 *Nat. Genet. ;* [3] Hop et al. 2022 *Sci. Transl. Med. ;* [4] Gagliano et al. 2016 *Ann. Clin. Transl. Neurol.;* [5] Kim et al. 2021 *Nat. Comm. ;* [6] Herrick & Tansey 2021 *npj Parkinson's Disease.*

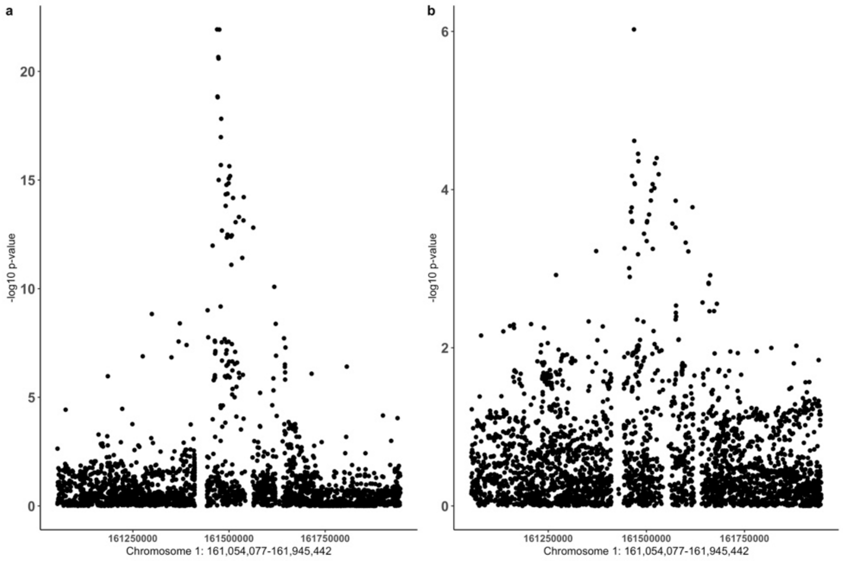

Supplementary Figure 2. Regional plots of GWAS, encompassing the genomic region chr1: 161,054,077-161,945,442, for which a significant correlation was observed between (a) UC and (b) PD. Each data point corresponds to a genomic variant.

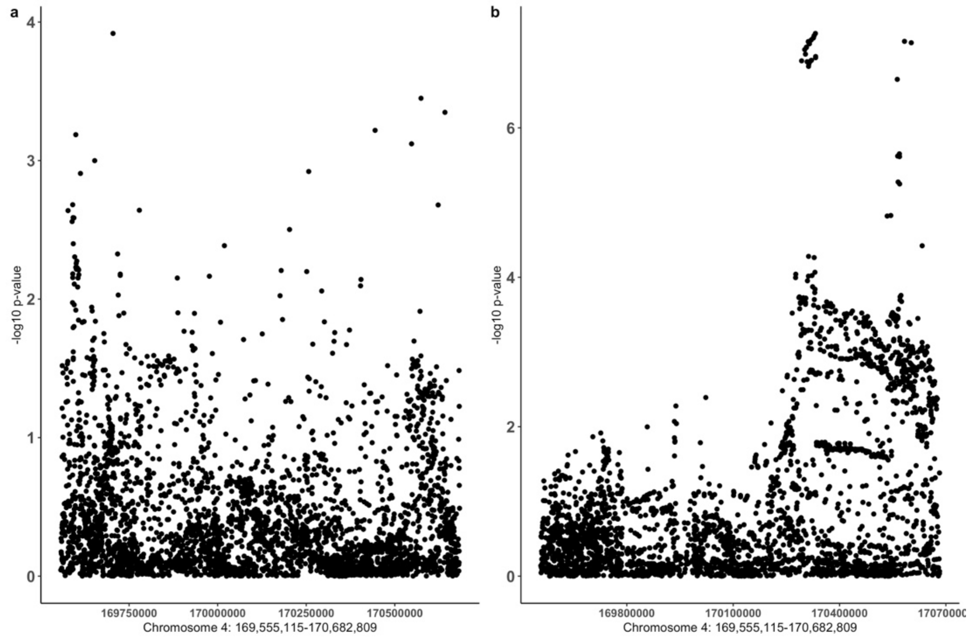

Supplementary Figure 3. Regional plots of GWAS, encompassing the genomic region chr4: 169,555,115-170,682,809, for which a significant correlation was observed between (a) CD and (b) ALS. Each data point corresponds to a genomic variant.

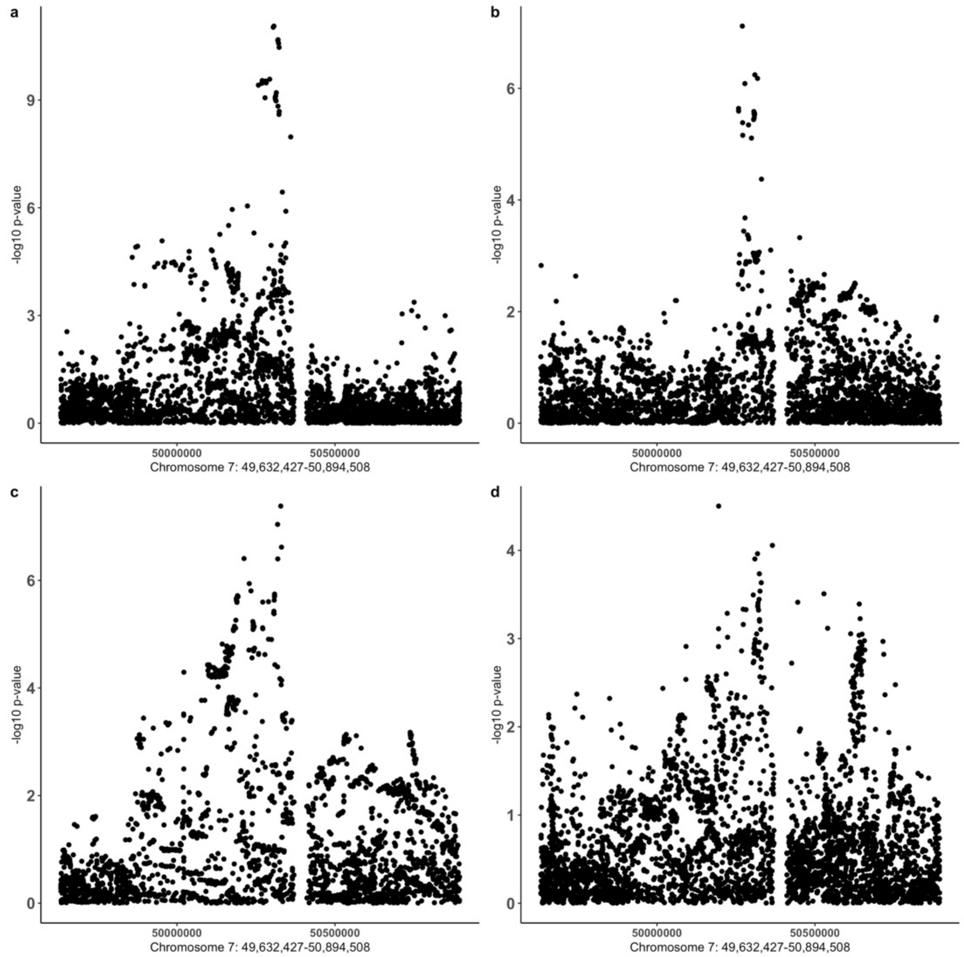

Supplementary Figure 4. Regional plots of GWAS, encompassing the genomic region chr7: 49,632,427-50,894,508, for which a significant correlation was observed between (a) CD, (b) AD, (c) MS and (d) UC. Each data point corresponds to a genomic variant.

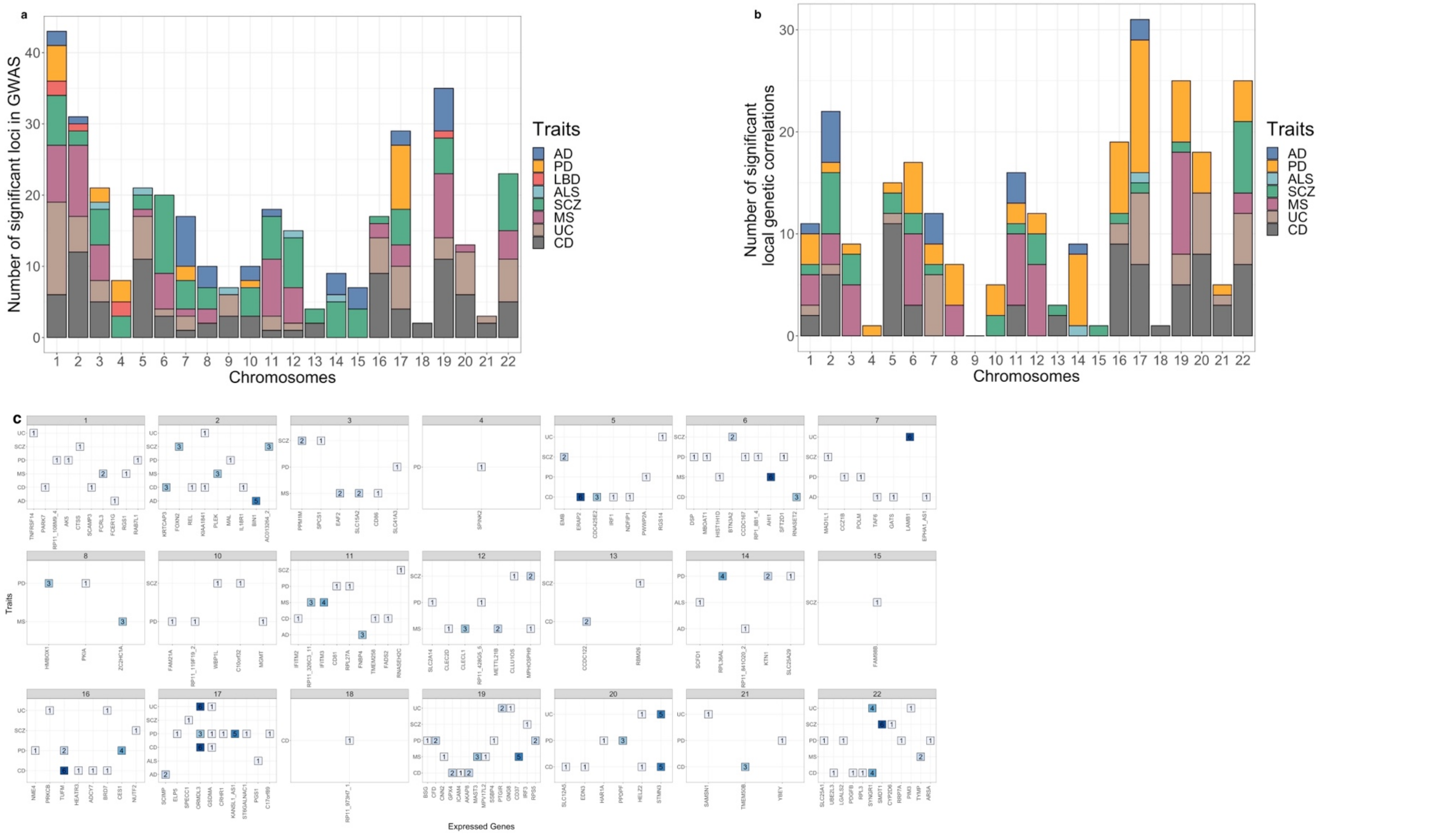

Supplementary Figure 5. Overview of the significant regional correlations (FDR < 0.01) between diseases and genes expressed across seven immune cell types, excluding the Human Leukocyte Antigen (HLA) region. (a) Distribution of the number of significant GWAS loci across autosomes across each tested disease. (b) Distribution of total number of significant correlations across autosomes for all cell types tested, stratified by disease. (c) Significant correlations between diseases and genes expressed. Each quadrant corresponds to an individual chromosome and the number inside each correlated pair of traits corresponds to the number of cell types in which a significant correlation was observed. AD = Alzheimer's disease, PD = Parkinson's disease, ALS = Amyotrophic lateral sclerosis, SCZ = Schizophrenia, MS = Multiple sclerosis, UC = Ulcerative colitis, CD = Crohn's disease.

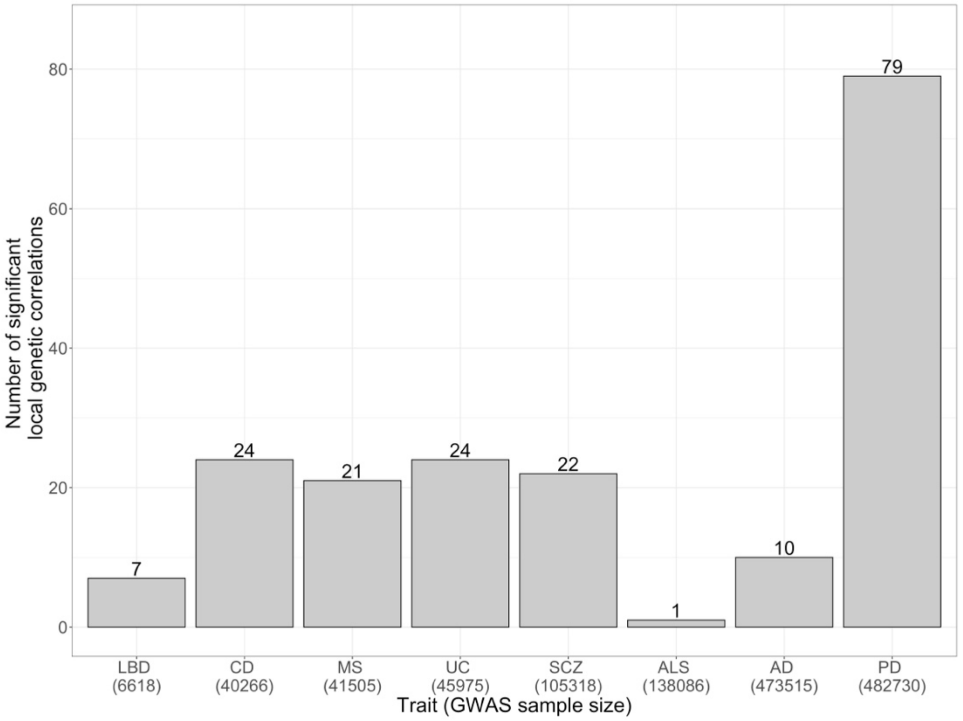

Supplementary Figure 6. Significant regional correlations (FDR < 0.01) between diseases and protein levels. Traits on the x-axis are ordered by ascending GWAS sample size (N cases + N controls).

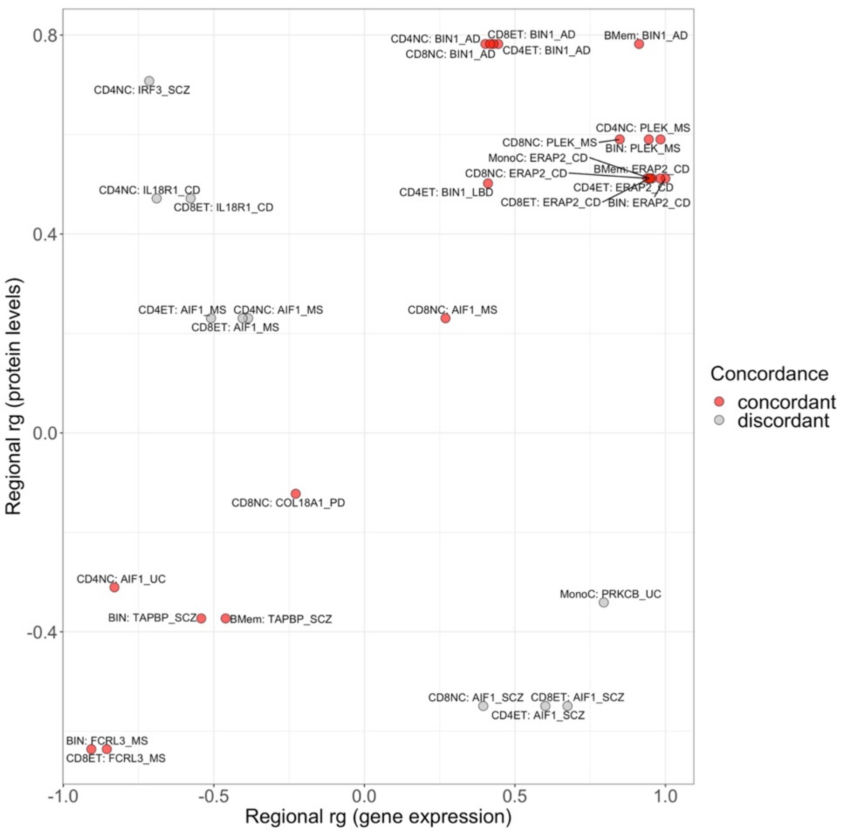

Supplementary Figure 7. Scatterplot of the regional genetic correlations (rg) for concordant and discordant nominally significant signals between correlations performed with gene expression levels (x-axis) and with protein levels (y-axis) across diseases tested. We defined concordant signals as those for which the rg between both disease-gene expression levels and the corresponding disease-protein levels was in the same direction. The cell type (in which the gene is expressed), the gene symbol and the disease are displayed for each datapoint. BIN = naïve B cells; BMem = Memory B cells; CD4ET = CD4+ effector memory T cells; CD4NC = CD4+ naïve T cells; CD8ET = CD8+ effector memory T cells; CD8NC = CD8+ naïve T cells; MonoC = classical monocyte.

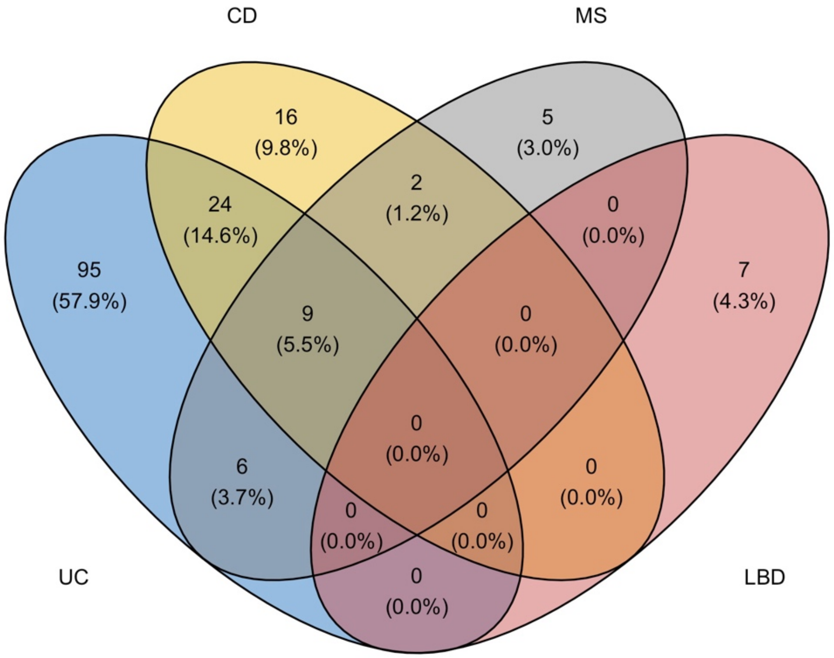

Supplementary Figure 8. Overlap of significant (FDR < 0.05) Gene Ontology (GO) biological processes among diseases. The GO biological processes displayed here are listed in Supplementary File 1. CD = Crohn's disease; LBD = Lewy body dementia; MS = Multiple sclerosis; UC = Ulcerative colitis.

Regional correlations across GWAS datasets → 2,495 LD blocks across the autosomes → 389 tested LD blocks (with ≥ 1 genome-wide significant variant in ≥ 1 GWAS) → Number of LD blocks (N) with univariate signal per GWAS dataset:

GWAS	N
AD	46
ALS	46
CD	134
FTD	3
LBD	38
MS	85
PD	338
SCZ	184
UC	110

Regional correlations with sc-eQTLs

Total number of genes with genome-wide significant QTLs per cell-type (N):

Cell-type	N
Monocytes	196
CD4+ effector T cells	543
CD4+ naïve T cells	2,089
CD8+ effector T cells	1,018
CD8+ naïve T cells	930
Naïve B cells	502
Memory B cells	383

Total number of genes for each cell-type with univariate signal across GWAS datasets:

Cell-type	AD	ALS	CD	LBD	MS	PD	SCZ	UC
Monocytes	9	1	9	0	5	49	10	7
CD4+ effector T cells	6	4	17	1	14	115	14	9
CD4+ naïve T cells	3	7	24	2	20	235	15	12
CD8+ effector T cells	15	8	27	1	25	241	32	18
CD8+ naïve T cells	14	5	30	1	22	201	32	18
Naïve B cells	11	4	16	1	21	113	24	13
Memory B cells	9	4	9	1	14	87	17	7

Regional correlations with pQTLs

1,553 proteins with genome-wide significant QTLs

Total number of proteins with univariate signal across GWAS datasets:

AD	ALS	CD	LBD	MS	PD	SCZ	UC	FTD
44	24	127	39	91	1,374	76	87	1

Supplementary Figure 9. Flowchart of the number of tests included in each of the regional genetic correlation analyses.

Supplementary Note 1

Validation of significant regional genetic correlations with gene expression and protein levels, in AD and PD datasets without proxy cases

We aimed at verifying that our results involving Alzheimer's disease (AD) and Parkinson's disease (PD) are not primarily driven by possible spurious effects of the inclusion of proxy cases in the GWAS samples. To do so, we performed regional genetic correlations with LAVA between the significant signals (FDR < 0.01) identified between AD or PD and gene expression or protein levels, using as input the AD or PD GWAS datasets without proxy cases [1,2]. The aim was to compare results with or without the use of proxy cases for the AD and PD GWAS datasets.

Genome-wide genetic correlations without proxy cases

We computed genome-wide genetic correlations between pairs of traits, including the AD and PD datasets without proxy cases (Supplementary Figure 10). These GWASes exhibit a perfect global genetic correlation with the corresponding GWAS including proxy cases (AD: r_g = 1.07 p-value = 9.86e-82; PD: r_g = 1.04, p-value = 0). By using a Bonferroni-corrected p-value threshold (p-value < 0.0014), we identified the same significant correlations as in the main analysis, which used the AD and PD GWAS association results that included proxy cases. Previous studies have highlighted genome-wide genetic correlations among neurodegenerative diseases, such as between LBD and AD, for which we did not observe a significant correlation in our main analysis (r_g = 0.33; p-value = 0.1324), and between AD and PD, for which we observed a nominal correlation when using the datasets with proxy cases (r_g = 0.23; p-value = 0.0096). When using the AD dataset without proxy cases, we observed nominally significant genetic correlations between LBD and AD (r_g = 0.69, p-value = 4.1e-03). For AD and PD, the correlation remained non-significant when using both the AD and PD datasets without proxies (rg = 0.12, p-value = 0.2).

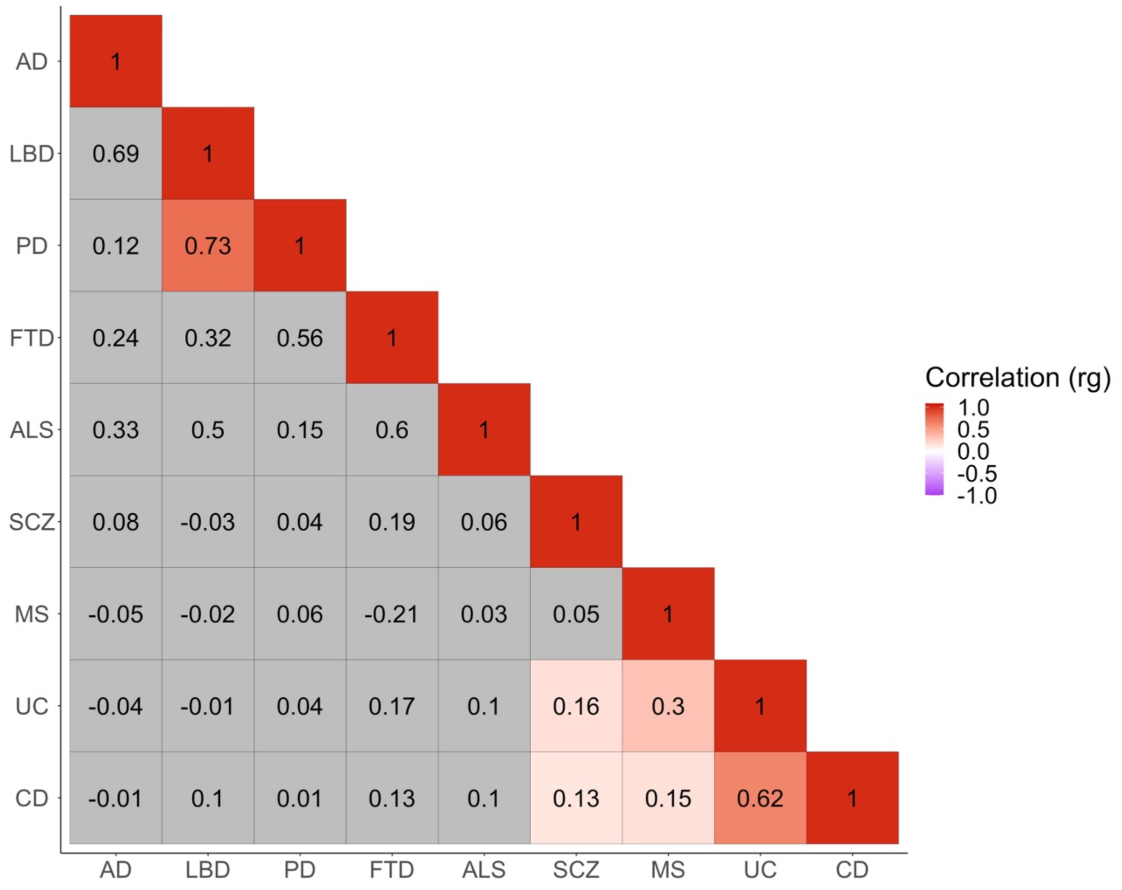

Supplementary Figure 10. Genome-wide genetic correlations (r_g) across GWAS traits without AD or PD proxy cases. Significant positive correlations (Bonferroni corrected p-value < 0.0014) are highlighted in shades of red. AD = Alzheimer's disease; LBD = Lewy body dementia; PD = Parkinson's disease; FTD = frontotemporal dementia; ALS = amyotrophic lateral sclerosis; SCZ = schizophrenia; MS = multiple sclerosis; UC = ulcerative colitis; CD = Crohn's disease.

Validation of significant correlations with gene expression levels

In the primary analysis with proxy cases, we observed 30 significant correlations between AD and genes expressed across all seven cell types tested, encompassing 14 unique genes, including the HLA region. We reran LAVA across 21 out of the 30 significant AD correlations, given that nine of

them did not have significant univariate signal in the AD GWAS without proxy cases (Supplementary Table 3; Supplementary Figure 11a). By using a Bonferroni p-value threshold for multiple testing (p-value < 0.05/30 tests performed for AD), we observed 12 significant correlations (p-value < 0.0016) and eight nominally significant correlations (p-value < 0.05) in the AD replication analysis for gene expression levels (Supplementary Figure 11a). One significant correlation in the primary analysis did not replicate, implicating the expression of the gene *RP11_841O20_2* (Ensembl ID: ENSG00000258757) in CD4+ effector T cells (r_g = 0.445; p-value = 0.125). However, the direction of effect was concordant in all tests (Spearman's correlation = 0.99; p-value = 2.2e-16) (Supplementary Figure 11a).

In the primary correlation analysis between PD and gene expression levels, we observed 78 significant correlations across all seven cell types tested, encompassing 53 unique genes. We were able to perform the replication analysis for 10 of the primary significant correlations, given that 68 did not have sufficient univariate signal in the PD GWAS without proxy cases (Supplementary Table 3; Supplementary Figure 11b). We applied a Bonferroni p-value threshold accounting for 78 significant correlations observed in the primary analysis (p-value < 0.05/78 tests). There were five significant correlations (p-value < 0.0006) and five nominally significant correlations (p-value < 0.05) in the PD validation analysis for gene expression levels, all with the same direction of effect (Spearman's correlation = 0.80; p-value = 0.006) (Supplementary Figure 11b).

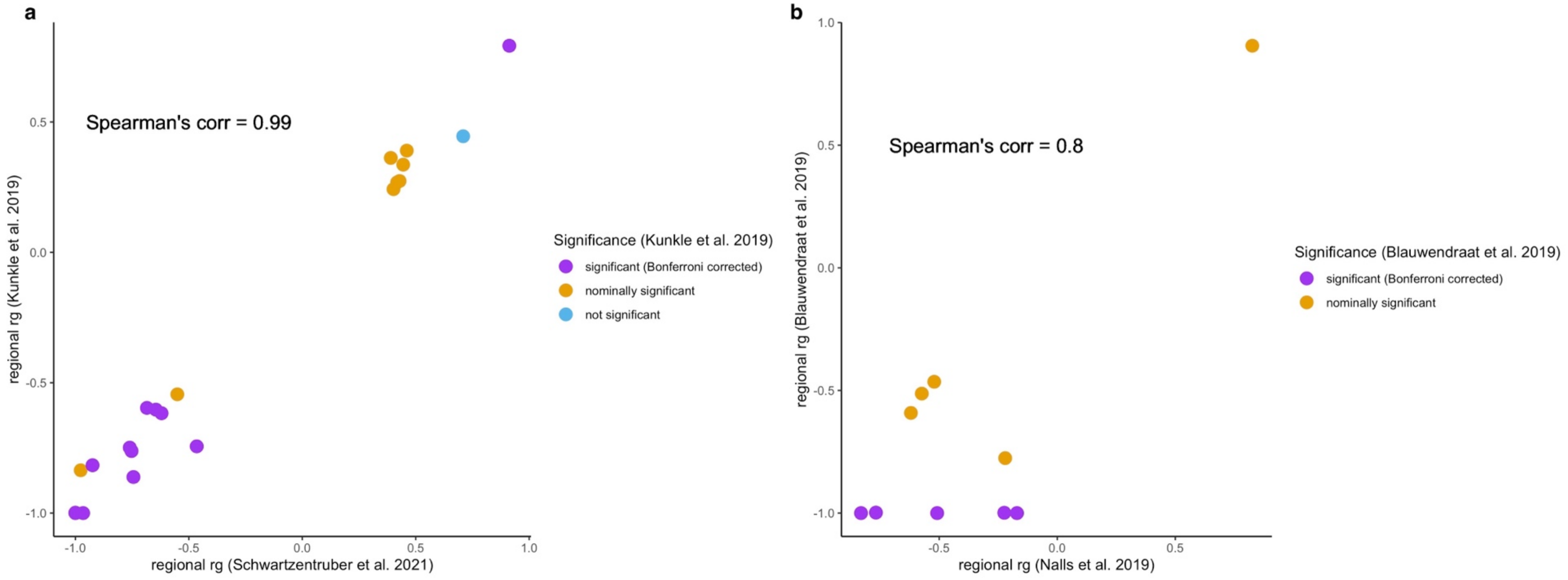

Supplementary Figure 11. Scatter plots between the primary (i.e., with proxy cases) and replication (i.e., without proxy cases) regional genetic correlation analyses between (a) AD and gene expression levels, and (b) PD and gene expression levels.

Validation of significant correlations with protein levels

In the primary analysis with proxy cases, there were 10 significant correlations between AD and protein levels. From these, we performed the validation analysis at eight loci, given that two of them did not have sufficient univariate signal (Supplementary Table 4; Supplementary Figure 12a). After applying a Bonferroni p-value threshold for multiple testing (p-value < 0.05/10 tests), we observed seven significant correlations in the validation without proxies (p-value < 0.005) and one nominally significant correlation (p=value < 0.05). Additionally, the direction of effect was concordant between the primary and validation analyses (Spearman's correlation = 0.99; p-value = 1.589e-06) (Supplementary Figure 12a).

In the primary correlations between PD and protein levels, there were 79 significant correlations. We performed the validation analysis only for six of the significant correlations that had sufficient univariate signal in the PD GWAS without proxy cases (Supplementary Table 4; Supplementary Figure 12b). By applying a Bonferroni p-value threshold to account for the number of significant correlations in the primary analysis (p-value < 0.05/79 tests), we observed three significant correlations (p-value < 0.0006) and three nominally significant correlations (p-value < 0.05), all of which had a concordant direction of effect (Spearman's correlation = 0.98; p-value = 0.0004) (Supplementary Figure 12b).

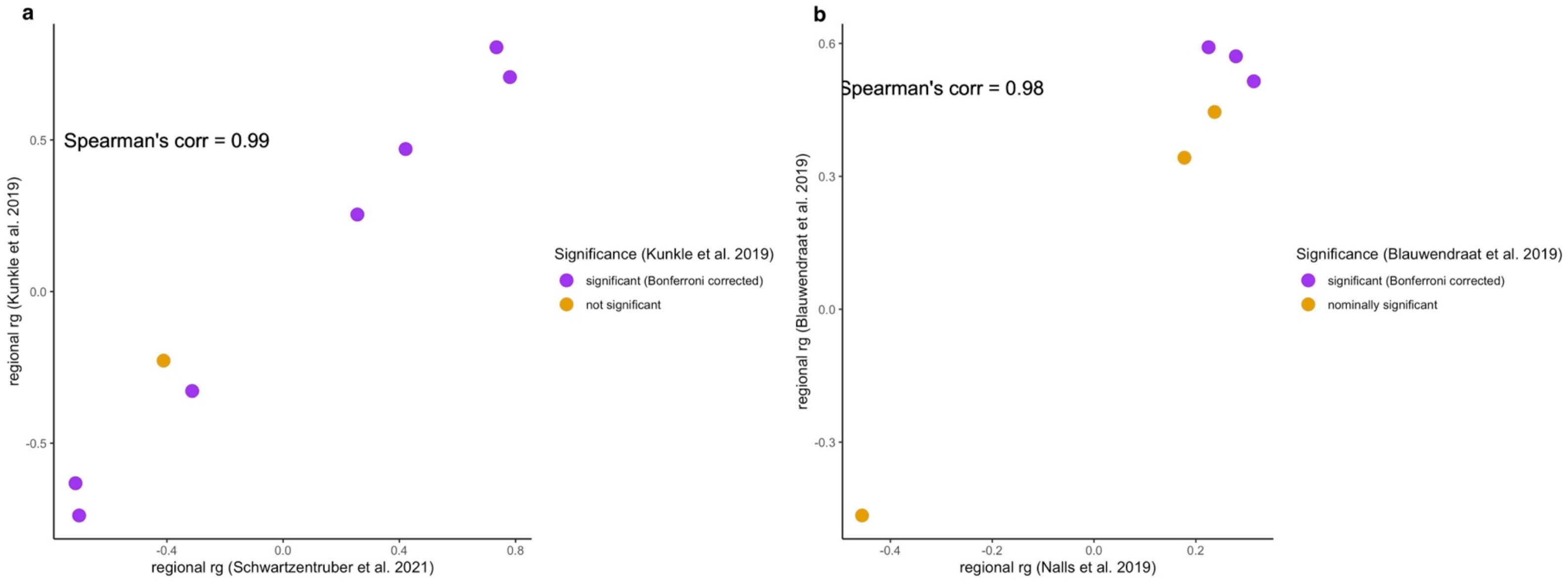

Supplementary Figure 12. Scatter plots between the primary (i.e., with proxy cases) and validation (i.e., without proxy cases) regional genetic correlation analyses between (a) AD and protein levels, and (b) PD and protein levels.

Supplementary Table 3. Validation of significant regional genetic correlations between AD or PD (using GWAS with proxy cases) and gene expression levels, using GWAS datasets without proxy cases.

Disease	Gene Expressed	Cell type	Results (with proxy cases)		Validation (without proxy cases)	
			r_g	p-value	r_g	p-value
AD	*BIN1*	BMem	0.912	7.10E-06	0.792	7.94E-04
AD	*BIN1*	CD4ET	0.445	4.40E-04	0.336	0.015
AD	*BIN1*	CD4NC	0.402	2.82E-07	0.242	0.011
AD	*BIN1*	CD8ET	0.429	1.68E-07	0.274	4.93E-03
AD	*BIN1*	CD8NC	0.418	3.97E-05	0.268	0.020
AD	*HLA_DRB1*	BIN	-0.465	1.12E-03	-0.744	3.09E-04
AD	*HLA_DQA1*	CD8ET	-1	6.70E-09	-0.998	3.19E-04
AD	*HLA_DQA1*	MonoC	-0.551	9.68E-04	-0.544	0.022
AD	*HLA_DQB1*	CD8ET	0.39	3.07E-03	0.362	0.032
AD	*HLA_DQB1*	MonoC	0.46	8.82E-05	0.39	0.029
AD	*HLA_DQA2*	BIN	-0.685	2.22E-10	-0.597	1.98E-04
AD	*HLA_DQA2*	BMem	-0.62	1.34E-06	-0.617	2.11E-04
AD	*HLA_DQA2*	CD8ET	-0.645	1.71E-12	-0.603	2.92E-06
AD	*HLA_DQA2*	CD8NC	-0.744	4.54E-09	-0.862	2.72E-05
AD	*HLA_DQA2*	MonoC	-0.966	1.41E-10	-1	7.72E-06
AD	*GATS*	BIN	-0.977	3.39E-06	-0.836	0.011
AD	*EPHA1_AS1*	MonoC	-1	3.06E-09	-1	2.50E-07
AD	*FNBP4*	BMem	-0.924	3.32E-04	-0.816	6.49E-04
AD	*FNBP4*	CD8ET	-0.753	2.18E-03	-0.762	8.89E-04
AD	*FNBP4*	CD8NC	-0.76	2.03E-03	-0.749	1.58E-03
AD	*RP11_841O20_2*	CD4ET	0.709	1.82E-03	0.445	0.125
PD	*RAB7L1*	CD4NC	0.826	1.06E-03	0.905	8.22E-04
PD	*HLA_DRB5*	MonoC	-0.221	6.91E-04	-0.776	2.44E-03

PD	*HMBOX1*	BIN	-0.574	3.19E-04	-0.513	1.00E-03
PD	*HMBOX1*	BMem	-0.62	8.75E-04	-0.592	9.93E-04
PD	*HMBOX1*	CD8NC	-0.521	3.17E-03	-0.465	6.33E-03
PD	*CRHR1*	CD8NC	-0.509	4.29E-04	-1	3.18E-06
PD	*KANSL1_AS1*	BIN	-0.225	1.24E-05	-0.999	2.44E-11
PD	*KANSL1_AS1*	BMem	-0.171	1.73E-03	-1	5.51E-11
PD	*KANSL1_AS1*	CD8ET	-0.831	1.34E-39	-1	6.51E-08
PD	*KANSL1_AS1*	CD8NC	-0.768	1.13E-25	-0.998	1.73E-09

BIN = naïve B cells; BMem = Memory B cells; CD4ET = CD4+ effector memory T cells; CD4NC = CD4+ naïve T cells; CD8ET = CD8+ effector memory T cells; CD8NC = CD8+ naïve T cells; MonoC = classical monocytes.

Supplementary Table 4. Validation of significant regional genetic correlations between AD or PD (using GWAS with proxy cases) and protein levels, using GWAS datasets without proxy cases.

**Disease**	**Protein gene symbol**	**Results (with proxy cases)**		**Validation (without proxy cases)**	
		$\mathbf{r_g}$	**p-value**	$\mathbf{r_g}$	**p-value**
AD	*CR1*	0.734	6.00E-20	0.806	4.00E-15
AD	*BIN1*	0.781	2.90E-19	0.707	3.88E-12
AD	*C2*	-0.702	1.09E-07	-0.738	1.51E-05
AD	*ATF6B*	-0.411	1.37E-03	-0.228	0.149
AD	*TREM2*	-0.714	6.58E-07	-0.632	1.89E-04
AD	*PLCG2*	-0.313	3.03E-04	-0.328	2.59E-03
AD	*APOE*	0.421	1.08E-05	0.47	1.66E-06
AD	*APOC1*	0.255	3.51E-09	0.254	2.51E-08
PD	*FCGR2A*	0.237	6.02E-13	0.445	8.46E-04
PD	*CNTN2*	0.178	4.57E-04	0.342	6.83E-03
PD	*BST1*	0.314	2.87E-07	0.515	3.68E-04

PD	*TREML2*	0.279	1.72E-04	0.571	7.65E-05
PD	*PCBD1*	0.225	3.17E-04	0.591	1.34E-04
PD	*SIGLEC15*	-0.456	3.97E-05	-0.466	0.0176

Supplementary References

1. Kunkle, B. W. *et al.* Genetic meta-analysis of diagnosed Alzheimer's disease identifies new risk loci and implicates Aβ, tau, immunity and lipid processing. *Nat Genet* **51**, 414–430 (2019).
2. Blauwendraat, C. *et al.* Parkinson disease age at onset GWAS: defining heritability, genetic loci and alpha-synuclein mechanisms. *Mov Disord* **34**, 866–875 (2019).
