# Supplementary material for: Regional genetic correlations highlight relationships between neurodegenerative disease loci and the immune system
Source: Commun Biol. 2023 Jul 15;6:729. doi: 10.1038/s42003-023-05113-5 (PMC10349864; doi:10.1038/s42003-023-05113-5)
Supplement: Supplementary file 3 — Description of Additional Supplementary Files [file 42003_2023_5113_MOESM3_ESM.pdf]

Description of Additional Supplementary Files

File name: Supplementary Data 1
Description: Regional genetic correlations between GWAS diseases tested in the present study.

File name: Supplementary Data 2
Description: Significant results (FDR < 0.01) of regional genetic correlations between sc-eQTLs and diseases tested in the present study. Source data behind Figures 3 and 4.

File name: Supplementary Data 3
Description: Colocalization results between diseases and expressed genes across immune cell types for disease-cell type pairs with Posterior Probability, PP H4 ≥ 0.8, indicating evidence of a shared causal signal. Source data behind Figure 5.

File name: Supplementary Data 4
Description: Regional genetic correlations between diseases and gene expression levels, for which the implicated loci did not harbour genome-wide significant GWAS variants (p-value < 5E-8).

File name: Supplementary Data 5
Description: Significant results (FDR < 0.01) of regional genetic correlations between pQTLs and diseases tested in the present study.

File name: Supplementary Data 6
Description: Diseases with significant Gene Ontology biological processes.
